# Analytical Modeling for the Bending Resonant Frequency of Multilayered Microresonators with Variable Cross-Section

**DOI:** 10.3390/s110908203

**Published:** 2011-08-25

**Authors:** Agustín L. Herrera-May, Luz A. Aguilera-Cortés, Hector Plascencia-Mora, Ángel L. Rodríguez-Morales, Jian Lu

**Affiliations:** 1 Centro de Investigación en Micro y Nanotecnología, Universidad Veracruzana, Calzada Ruiz Cortines 455, 94292, Boca del Río, Veracruz, Mexico; 2 Depto. Ingeniería Mecánica, DICIS, Universidad de Guanajuato, Carr. Salamanca-Valle 3.5+1.8 km, Palo Blanco, 36885, Salamanca, Guanajuato, Mexico; E-Mail: aguilera@ugto.mx; 3 Depto. Ingeniería Molecular de Materiales, Centro de Física Aplicada y Tecnología Aplicada, UNAM, Boulevard Juriquilla 3001, 76230, Juriquilla, Queretaro, Mexico; E-mail: alrodriguez@fata.unam.mx; 4 Research Center for Ubiquitous MEMS and Micro Engineering (UMEMSME), National Institute of Advanced Industrial Science and Technology (AIST), Namiki 1-2-1, 305-8564, Tsukuba, Ibaraki, Japan; E-Mail: jian-lu@aist.go.jp

**Keywords:** bending resonant frequency, Euler-Bernoulli beam theory, Macaulay method, multilayered microresonator, Rayleigh’s method

## Abstract

Multilayered microresonators commonly use sensitive coating or piezoelectric layers for detection of mass and gas. Most of these microresonators have a variable cross-section that complicates the prediction of their fundamental resonant frequency (generally of the bending mode) through conventional analytical models. In this paper, we present an analytical model to estimate the first resonant frequency and deflection curve of single-clamped multilayered microresonators with variable cross-section. The analytical model is obtained using the Rayleigh and Macaulay methods, as well as the Euler-Bernoulli beam theory. Our model is applied to two multilayered microresonators with piezoelectric excitation reported in the literature. Both microresonators are composed by layers of seven different materials. The results of our analytical model agree very well with those obtained from finite element models (FEMs) and experimental data. Our analytical model can be used to determine the suitable dimensions of the microresonator’s layers in order to obtain a microresonator that operates at a resonant frequency necessary for a particular application.

## 1. Introduction

Multilayered microresonators fabricated using microelectromechanical systems (MEMS) have potential applications such as mixer-filters [1,2] and detection of mass [3,4], prostate-specific antigens [5], proteins [6], virus [7,8], chemical species [9], gas [10,11], and magnetic fields [12–14]. These microresonators have several advantages such as small size, high sensitivity, high resolution, low power consumption, and low cost in mass production [15,16]. They can be excited in the first bending resonant frequency through different types of sources such as magnetic, electrostatic, thermal, and piezoelectric [17]. Their frequency shift and deflections can also be monitored using capacitive, piezoelectric, piezoresistive or optical sensing techniques [18,19].

The microresonators are extremely sensitive to surface processes due to their large surface area to mass ratios [20]. For chemical and biological sensing, the surface of microresonators can be covered with a sensitive coating to absorb the chemical and biological species [21,22]. Several microresonators also use piezoelectric multilayers due to their advantages such as compact structure, low driving voltage, self-actuation and self-sensing capability, and easy integration with electronic circuits [23].

An important issue in the mechanical design process of a multilayered microresonator with variable cross-section is the correct determination of its fundamental resonant frequency. Another important parameter is the estimation of its deflection under an excitation load, which can be used to study its mechanical behavior. Theoretical models can predict the resonant frequency shift of microresonators caused by variations of their geometrical variables and mechanical properties. Lobontiu *et al*. developed lumped and distributed-parameter models for predicting the bending or torsion resonant frequencies of microresonators with variable cross section and serially connected [24–28]. They also obtained analytically the deflection of this microresonator type; nevertheless, their models do not consider multilayered microresonators. Dufour and Fadel [29] reported analytical expressions in order to calculate the resonant frequencies of thin parallelepiped microcantilevers formed by a structural layer and a sensitive coating. They calculated the bending resonant frequency of microcantilevers using the deflection expression of its bending resonance mode. Their models are suited to microcantilevers with a rectangular plate and a sensitive coating. Ferguson *et al*. [30] presented an analytical model for predicting the fundamental bending resonant frequency of a composite free-free microbeam, which is connected to four torsional anchors. In addition, they determined the fundamental torsional frequency of the composite anchors. However, these models are limited to composite free-free microbeams and anchors with uniform cross section. Della *et al*. [31] employed an analytical model in order to study the free vibration of delaminated multilayered beams with two fixed ends. They analyzed the fundamental frequency shift of clamped-clamped multilayer beams with single and double delaminations in terms of the following parameters: normalized axial stiffness, normalized bending stiffness, and the relative slenderness ratio. This model is limited to delaminated multilayered beams with uniform width. Sampath *et al*. [32] determined the resonant frequency of a two-layer hybrid microcantilever with uniform cross section, which is integrated by an elastic base and viscoelastic layer. They reported that the molecular absorption process affects the resonant frequency of the microcantilever due to that modifies the mass load and the viscoelastic properties of the sensitive coating. Li *et al*. [33,34] studied the nonlinear transverse vibrations of a clamped-clamped composite microbeam using a refined integro-partial differential model. This model considers a composite microbeam with stepwise axially varying properties, rectangular cross section, and non-flat equilibrium position (*i.e.*, a buckled beam). Based on this model, they estimated the first natural frequency of a clamped-clamped composite microbeam. For this, they employed a buckling factor that depends of the static compressive forces, stiffness, and geometric properties of the microbeam. Finally, they only reported the natural frequency of a multilayer microbeam with two clamped ends, which has a uniform width and a non-flat equilibrium position. Later, Li *et al*. [35] adopted a curved cross-section model to refine their previous buckling microbeam model [33,34]. This new model considers the cross-section curvature of the clamped-clamped composite microbeam induced by residual stress, which affects its structural stiffness and spatial response. Using this model, the analytical resonant frequencies of the clamped-clamped composite microbeam is closer to the experimental observations. However, it cannot be used for multilayered microresonators with variable width or serially connected multisegments. Pasini [36] developed a bending response model for multilayered microresonators considering their cross-section shape, symmetry and layers number, as well as their materials properties. However, his model does not consider different microbeam types connected in series. Edqvist *et al*. [37] established a general theoretical model for studying the quasi-static and dynamic electromechanical responses of piezoelectric multilayered microbeams with one fixed end. Their model was obtained through Euler-Bernoulli theory and only considers layers with uniform cross section. Herrera-May *et al*. [38,39] used theoretical models to estimate the first bending resonant frequency of microresonators integrated by an arrangement of microbeams with different cross-sections. All these models have important characteristics that can be useful in the design phase of some microresonators. However, several they have limitations to predict the resonant frequency of multilayered microresonators with variable cross-section. In order to overcome all these drawbacks, we present an analytical model for the first bending resonant frequency of multilayered microresonators with variable cross section and one fixed end. This model includes microresonators composed by an arrangement of different multilayered microbeam types. Furthermore, our model can be used to estimate the deflection expression of the proposed microresonators. The analytical model is based on the Rayleigh’s method, Macaulay’s functions, and Euler-Bernoulli beam theory. It does not consider the damping, residual stress and shear deformation of the microresonators.

This paper is organized as follows: in Section 2, we describe the analytical model for the bending resonant frequency of a multilayered microresonator with variable cross section. Next, in Section 3, this model is applied to two multilayered microresonators with mass sensing applications reported in the literature. The results of our analytical model are compared with those obtained from a simple cantilever model, FEMs, and experimental data. Finally, we presented our conclusions and proposed future research in Section 4.

## 2. Analytical Modeling

This section describes the analytical modeling to predict the first bending resonant frequency and deflection of multilayered microresonators with variable cross section. Rayleigh’s method can predict the first bending resonant frequency of structures with complex geometrical shapes considering energy conservation in the structures [40]. This method requires the deflection expression, bending stiffness, and mass per unit length of the structures.

The bending deflection response *y*(*x*,*t*) at a given point of a resonant structure with a harmonic motion can be determined as a product between a spatial function *y*(*x*) and a time-dependent one [27]. Thus, *y*(*x*,*t*) = *y*(*x*)sin(2π*ft*), where *t* is the time and *f* is the frequency.

For out-of plane bending in single and double clamped beams with length *L* and cross-section area *A*(*x*), the maximum potential energy (*U_m_*) and kinetic energy (*K_m_*) are determined by [41]:

(1) $$U_{m}=\frac{1}{2}\int_{0}^{L}\mathrm{EI}(x){\left(\frac{\partial^{2}y(x)}{{\partial x}^{2}}\right)}^{2}\mathrm{dx}$$

(2) $$K_{m}=\frac{{\left(2\pi f\right)}^{2}}{2}\int_{0}^{L}\rho A(x)y^{2}(x)\mathrm{dx}$$

where *E*, *I*, and *ρ* are the Young’s modulus, inertia moment, and density of the beam, respectively.

Based on Rayleigh’s method, the first bending resonant frequency (*f_r_*) of single and double clamped beams can be obtained assuming energy conservation (*U_m_* = *K_m_*) in the beams. By substituting Equations (1) and (2) into *U_m_* = *K_m_*, the *f_r_* is given by:

(3) $$f_{r}=\frac{1}{2\pi }{\left(\frac{\int_{0}^{L}\mathrm{EI}(x){\left(\frac{\partial^{2}y(x)}{{\partial x}^{2}}\right)}^{2}\mathrm{dx}}{\int_{0}^{L}\rho A(x)y^{2}(x)\mathrm{dx}}\right)}^{1/2}$$

**Figure 1.**
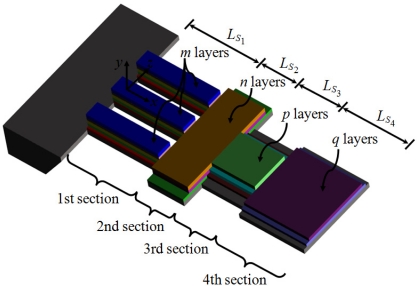
View of the multilayered microresonator with variable cross section proposed in this work.

**Figure 2.**
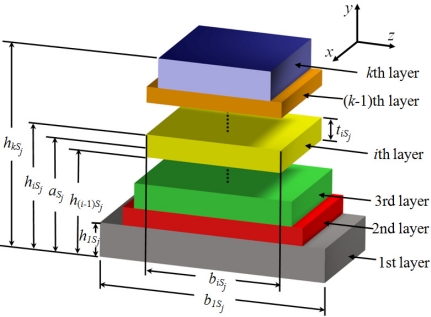
Geometrical nomenclature proposed for the *k*th layer located on the *j*th section of the proposed multilayered microresonator.

**Figure 3.**
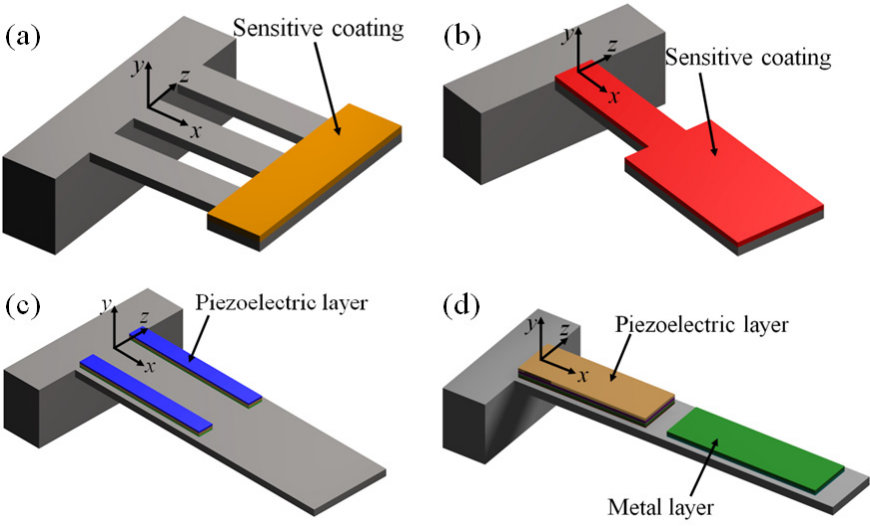
Examples of multilayered microresonators obtained from the proposed multilayered microresonator. These microresonators could are composed by silicon layers with (**a**–**b**) sensitive coating and (**c**–**d**) piezoelectric layers.

In order to use the Rayleigh’s method in a multilayered microresonator with variable cross section, we need to know its elastic centroid, bending stiffness and mass per unit length. We propose a multilayered microresonator with variable cross section, as shown in Figure 1. This microresonator is formed by layers of different materials and geometric shapes, which are symmetrically distributed with respect to the *xy*–plane. The microresonator is divided into four different sections, which contain *m*th, *n*th, *p*th, and *q*th layers, respectively. We employed the nomenclature *j*th section in order to describe each one of the four sections of the microresonator. Figure 2 shows the geometrical nomenclature proposed for *k*th layer placed over any of the *j*th section. In this case, *b_iSj_*, and *t_iSj_* represent the width and thickness of the *i*th layer located on the *j*th section. In addition, the parameter *h_iSj_* indicates the distance from the bottom plane of the first layer to the top plane of the *i*th layer placed over the *j*th section. Based on our multilayered microresonator, several microresonator designs used for detection of mass and gas can be obtained. Figure 3(a–d) illustrates the configurations of four different multilayered microresonators that are derived from our general-microresonator design. An analytical model for estimating the first bending resonant frequency of our general microresonator can help designers and researchers in the design phase of particular microresonators. For instance, an analytical model for a general multilayered microresonator can be used to know the effect of the materials and dimensions of the multilayers on the resonant frequency and deflection of particular microresonators.

**Figure 4.**
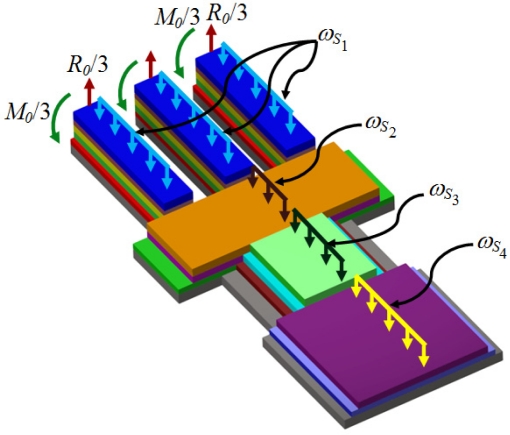
View of the load types acting on the proposed multilayered microresonator.

Figure 4 shows the reaction loads, bending moments, and uniformly distributed loads of the proposed microresonator.

For this case, *R*_0_ and M_0_ represent the total reaction load and bending moment at the fixed end of the microresonator. Furthermore, *ω_Sj_* indicates the uniformly distributed weight of all the layers located in the *j*th section. The analytical model developed in this work is based on the following assumptions:

- Multilayered microresonator is integrated by layers made of homogeneous and isotropic materials. The film mechanical properties of the different layers must be considered;
- The microresonator layers are symmetrically distributed with respect to *xy*–plane;
- The bending vibration of the microresonator occurs along of the *y*-axis and its vibration amplitude is smaller than its total thickness;
- The plane sections of layers do not deform, *i.e.*, transverse shear strain is neglected;
- The *xz*–plane of the coordinate system is located at the elastic centroide of the microresonator;
- The total length of the microresonator is greater than 10 times its total thickness. This is a necessary condition to use the Euler-Bernoulli beam theory;
- Damping and surface effects (e.g., surface energy, surface tension, surface relaxation, and surface reconstruction) are neglected;
- The residual stress in the microresonator is neglected;
- The nonlineality and viscosity of the layers are not considered;
- The undercut to the microresonator caused by etching processes is neglected.

For our microresonator, the elastic centroid *(a_Sj_*) in the *j*th section of the microresonator can be determined as [42]:

(4) $$\begin{array}{ll} a_{S_{j}} & =\frac{{\left(\mathrm{ES}\right)}_{S_{j}}}{{\left(\mathrm{EA}\right)}_{S_{j}}}=\frac{\iint_{A_{S_{j}}}E_{S_{j}}y_{S_{j}}(x)\mathrm{dydz}}{\iint_{A_{S_{j}}}E_{S_{j}}\mathrm{dydz}}\\ & =\frac{1}{2}\frac{\sum_{i=1}^{k}E_{{\mathrm{iS}}_{j}}b_{{\mathrm{iS}}_{j}}t_{{\mathrm{iS}}_{j}}\left(h_{{\mathrm{iS}}_{j}}+h_{(i-1)S_{j}}\right)}{\sum_{i=1}^{m}E_{{\mathrm{iS}}_{j}}b_{{\mathrm{iS}}_{j}}t_{{\mathrm{iS}}_{j}}} \end{array}$$

where *t_iSj_* = *h_iSj_* − *h*_(*i*−1)*Sj*_, the subscript *S_j_* is related to the *j*th section, the value *k* depends of the layers number located over each one of the four sections (*i.e.*, *k* can take either values *m*, *n*, *p* or *q*), *A_S_j__* symbolizes the domain occupied by the *j*th section, *y_Sj_* (*x*) is the deflection along of the *j*th section, *E_iSj_* is the Young’s modulus of the *i*th layer located in the *j*th section, *h*_(*i*−1)*Sj*_ is distance from the bottom plane of the first layer to the top plane of the (*i*-1)th layer placed over the *j*th section, (*ES*)*_S_j__* is the elastic modulus weighted first moment of area and (*EA*)*_S_j__* the elastic modulus weighted area. For the first section, *b*_*iS*1_ considers the overall width of the *i*th layer (*i.e.*, *b*_*iS*1_ includes the sum of the width of the three layers located on the same distance *h_iS1_*, which have the same material). In addition, the term *h_0Sj_* = 0.

The effective bending stiffness *(EI_z_*)*_Sj_* in the *j*th section of the multilayered microresonator can be calculated as:

(5) $$\begin{array}{ll} {\left({\mathrm{EI}}_{z}\right)}_{S_{j}} & =\sum_{i}^{k}{\left(E_{i}I_{\mathrm{zi}}\right)}_{S_{j}}=\iint_{A_{S_{j}}}E_{S_{j}}y_{S_{j}}(x)\mathrm{dy}\\ & =\frac{1}{3}\sum_{i=1}^{k}E_{{\mathrm{iS}}_{j}}b_{{\mathrm{iS}}_{j}}\left[{\left(h_{{\mathrm{iS}}_{j}}-a_{S_{j}}\right)}^{3}-{\left(h_{(i-1)S_{j}}-a_{S_{j}}\right)}^{3}\right] \end{array}$$

The maximum potential energy (*U_mL_*) and kinetic energy (*K_mL_*) of the multilayered microresonator can be obtained as:

(6) $$\begin{array}{lll} U_{\mathrm{mL}} & = & \frac{1}{2}{\left({\mathrm{EI}}_{z}\right)}_{S_{1}}\int_{0}^{L_{S_{1}}}{\left(\frac{\partial^{2}y_{S_{1}}(x)}{{\partial x}^{2}}\right)}^{2}\mathrm{dx}+\frac{1}{2}{\left({\mathrm{EI}}_{z}\right)}_{S_{2}}\int_{L_{S_{1}}}^{L_{S_{12}}}{\left(\frac{\partial^{2}y_{S_{2}}(x)}{{\partial x}^{2}}\right)}^{2}\mathrm{dx}\\ & & +\frac{1}{2}{\left({\mathrm{EI}}_{z}\right)}_{S3}\int_{L_{S_{12}}}^{L_{S_{123}}}{\left(\frac{\partial^{2}y_{S3}(x)}{{\partial x}^{2}}\right)}^{2}\mathrm{dx}+\frac{1}{2}{\left({\mathrm{EI}}_{z}\right)}_{S_{4}}\int_{L_{S_{123}}}^{L_{S_{1234}}}{\left(\frac{\partial^{2}y_{S_{4}}(x)}{{\partial x}^{2}}\right)}^{2}\mathrm{dx} \end{array}$$

(7) $$\begin{array}{lll} \frac{K_{\mathrm{mL}}}{\lambda^{2}} & = & \frac{1}{2}\left(\sum_{i=1}^{m}\rho_{{\mathrm{iS}}_{1}}b_{{\mathrm{iS}}_{1}}h_{{\mathrm{iS}}_{1}}\right)\int_{0}^{L_{S_{1}}}{\left(y_{S_{1}}(x)\right)}^{2}\mathrm{dx}+\frac{1}{2}\left(\sum_{i=1}^{n}\rho_{{\mathrm{iS}}_{2}}b_{{\mathrm{iS}}_{2}}h_{{\mathrm{iS}}_{2}}\right)\int_{L_{S_{1}}}^{L_{S_{12}}}{\left(y_{S_{2}}(x)\right)}^{2}\mathrm{dx}\\ & & +\frac{1}{2}\left(\sum_{i=1}^{p}\rho_{{\mathrm{iS}}_{3}}b_{{\mathrm{iS}}_{3}}h_{{\mathrm{iS}}_{3}}\right)\int_{L_{S_{12}}}^{L_{S_{123}}}{\left(y_{S_{3}}(x)\right)}^{2}\mathrm{dx}+\frac{1}{2}\left(\sum_{i=1}^{q}\rho_{{\mathrm{iS}}_{4}}b_{{\mathrm{iS}}_{4}}h_{{\mathrm{iS}}_{4}}\right)\int_{L_{S_{123}}}^{L_{S_{1234}}}{\left(y_{S_{4}}(x)\right)}^{2}\mathrm{dx} \end{array}$$

where *L*_*S*12_ = *L*_*S*1_ + *L*_*S*2_, *L*_*S*123_ = *L*_*S*1_ + *L*_*S*2_ + *L*_*S*3_, *L*_*S*1234_ = *L*_*S*1_ + *L*_*S*2_ + *L*_*S*3_+ *L*_*S*4_, and *λ* = 2π*f*.

Using the Rayleigh’s method, the first bending resonant frequency of the multilayered microresonator is obtained as:

(8) $$f_{r}=\frac{1}{2\pi }{\left(\frac{U_{\mathrm{mL}}}{K_{\mathrm{mL}}/\lambda^{2}}\right)}^{1/2}$$

Equations (6–8) show a dependence of the bending resonant frequency with respect to the deflection *y(x*), mechanical properties (Young’s modulus and density) and geometrical parameters of the layers from each section of the resonant microestructure. Any variation of these factors will cause a change in its resonant frequency. For the case of mass and gas sensing, the particles deposited on the surface of a microresonator can affect its bending resonant frequency due to changes of its mass and stiffness. This frequency shift is used to detect the amount and type of particles deposited on the microresonator surface. For the case of small particles deposited on a concentrated region of the microresonator surface, the resonant frequency shift can be estimated only considering the kinetic energy variation. In order to determine this energy shift, the mass added by the particles must be included into Equation (7). For instance, if the mass of the particles is considered as a small concentrated mass load (*m_add_*) located to a distance *x_add_* of the microresonator’s fixed end, then the term *m_add_y*^2^(*x_add_*)/2 must be summed into Equation (7). For more information of this topic we recommended the work developed by Dong *et al*. [11]. We do not include the study of the resonant frequency shift of the microresonator.

The deflection *y_Sj_* (*x*) in each one of the four sections of the microresonator are obtained using Euler-Bernoulli beam theory and Macaulay’s method [43]. This method is suitable to describe several load types acting on structures with variable cross section [44]. For this, it employs functions whose nomenclature considers a bracket notation 〈*x* − *c*〉*^ε^*, since these functions have the zero value for *x* < *c* and (*x* − *c*)*^ε^* for *x* ≥ *c* [45]. The value of superscript *ε* depends of the load type acting on structure. For instance, *ε =* 0 for a uniformly distributed load, *ε =* −1 for a concentrated load, and *ε* = −2 for a bending moment.

Thus, the deflection in each one of the four sections of proposed microresonator are determined from:

(9a) $${\left({\mathrm{EI}}_{z}\right)}_{S_{1}}\frac{\partial^{2}y_{S_{1}}(x)}{\partial^{2}x}=M_{S_{1}}(x)\;\;0<x<L_{S_{1}}$$

(9b) $${\left({\mathrm{EI}}_{z}\right)}_{S_{2}}\frac{\partial^{2}y_{S_{2}}(x)}{\partial^{2}x}=M_{S_{2}}(x)\;\;L_{S_{1}}<x<L_{S_{12}}$$

(9c) $${\left({\mathrm{EI}}_{z}\right)}_{S_{3}}\frac{\partial^{2}y_{S_{3}}(x)}{\partial^{2}x}=M_{S_{3}}(x)\;\;L_{S_{12}}<x<L_{S_{123}}$$

(9d) $${\left({\mathrm{EI}}_{z}\right)}_{S_{4}}\frac{\partial^{2}y_{S_{4}}(x)}{\partial^{2}x}=M_{S_{4}}(x)\;\;L_{S_{123}}<x<L_{S_{1234}}$$

where *M_Sj_* is the bending moment in the *j*th section of the multilayered microresonator, which can be determined through Macaulay’s functions.

The deflection *y_Sj_*(*x*) of our microresonator must satisfy the following boundary conditions:

(10) $$\begin{array}{cc} y_{S_{1}}(0)=0 & \frac{\partial y_{S_{1}}(0)}{\partial x}=0\\ y_{S_{1}}(L_{S_{1}})=y_{S_{2}}(L_{S_{1}}) & \frac{\partial y_{S_{1}}(L_{S_{1}})}{\partial x}=\frac{\partial y_{S_{2}}(L_{S_{1}})}{\partial x}\\ y_{S_{2}}(L_{S_{12}})=y_{S_{3}}(L_{S_{12}}) & \frac{\partial y_{S_{2}}(L_{S_{12}})}{\partial x}=\frac{\partial y_{S_{3}}(L_{S_{12}})}{\partial x}\\ y_{S_{3}}(L_{S_{123}})=y_{S_{4}}(L_{S_{123}}) & \frac{\partial y_{S_{3}}(L_{S_{123}})}{\partial x}=\frac{\partial y_{S_{4}}(L_{S_{123}})}{\partial x} \end{array}$$

Using Macaulay’s method, we find the total load function *F*(*x*) of the microsensor. It can be described as [43]:

(11) $$\begin{array}{lll} F(x) & = & -M_{0}{\langle x-0\rangle }^{-2}+R_{0}{\langle x-0\rangle }^{-1}-\omega_{S_{1}}{\langle x-0\rangle }^{0}+\omega_{S_{1}}{\langle x-L_{S_{1}}\rangle }^{0}-\omega_{S_{2}}{\langle x-L_{S_{1}}\rangle }^{0}+\omega_{S_{2}}{\langle x-L_{S_{12}}\rangle }^{0}\\ & & -\omega_{S_{3}}{\langle x-L_{S_{12}}\rangle }^{0}+\omega_{S_{3}}{\langle x-L_{S_{123}}\rangle }^{0}-\omega_{S_{4}}{\langle x-L_{S_{123}}\rangle }^{0}+\omega_{S_{4}}{\langle x-L_{S_{1234}}\rangle }^{0} \end{array}$$

with

(12) $$R_{0}=\sum_{i=1}^{4}\omega_{S_{j}}L_{S_{j}}$$

(13) $$M_{0}=\sum_{i=1}^{4}\omega_{S_{j}}L_{S_{j}}\left(\frac{1}{2}L_{S_{j}}+L_{S_{j-1}}+L_{S_{j-2}}+L_{S_{j-3}}\right)$$

(14) $$\omega_{S_{j}}=\sum_{i=1}^{k}\rho_{{\mathrm{iS}}_{j}}gb_{{\mathrm{iS}}_{j}}t_{{\mathrm{iS}}_{j}}$$

where *ω_Sj_* represents the weight per unit length in the *j*th section and *g* is the gravitational acceleration. For the first section, *b*_*iS*1_ considers the overall width of the all layers located in *h*_*iS*1_ (e.g., if there are three layers made with the same material located to a distance *h*_*iS*1_, then *b*_*iS*1_ includes the sum of each width of the layers).

The shear load function *V*(*x*) is obtained by integrating Equation (11) with respect to *x* and using the integration rules of the Macaulay’s functions [43]. The function *V*(*x*) is given by:

(15) $$\begin{array}{lll} V(x) & = & -M_{0}{\langle x-0\rangle }^{-1}+R_{0}{\langle x-0\rangle }^{0}-\omega_{S_{1}}{\langle x-0\rangle }^{1}+\omega_{S_{1}}{\langle x-L_{S_{1}}\rangle }^{1}-\omega_{S_{2}}{\langle x-L_{S_{1}}\rangle }^{1}+\omega_{S_{2}}{\langle x-L_{S_{12}}\rangle }^{1}\\ & & -\omega_{S_{3}}{\langle x-L_{S_{12}}\rangle }^{1}+\omega_{S_{3}}{\langle x-L_{S_{123}}\rangle }^{1}-\omega_{S_{4}}{\langle x-L_{S_{123}}\rangle }^{1}+\omega_{S_{4}}{\langle x-L_{S_{1234}}\rangle }^{1}+C_{1} \end{array}$$

Integrating Equation (15) with respect to *x*, the bending moment function *M*(*x*) is determined as:

(16) $$\begin{array}{lll} M(x) & = & -M_{0}{\langle x-0\rangle }^{0}+R_{0}{\langle x-0\rangle }^{1}-\frac{1}{2}\omega_{S_{1}}{\langle x-0\rangle }^{2}+\frac{1}{2}\omega_{S_{1}}{\langle x-L_{S_{1}}\rangle }^{2}-\frac{1}{2}\omega_{S_{2}}{\langle x-L_{S_{1}}\rangle }^{2}+\frac{1}{2}\omega_{S_{2}}{\langle x-L_{S_{12}}\rangle }^{2}\\ & & -\frac{1}{2}\omega_{S_{3}}{\langle x-L_{S_{12}}\rangle }^{2}+\frac{1}{2}\omega_{S_{3}}{\langle x-L_{S_{123}}\rangle }^{2}-\frac{1}{2}\omega_{S_{4}}{\langle x-L_{S_{123}}\rangle }^{2}+\frac{1}{2}\omega_{S_{4}}{\langle x-L_{S_{1234}}\rangle }^{2}+C_{1}x+C_{2} \end{array}$$

where *C*_1_ and *C*_2_ are constants calculated through boundary conditions (*V*(0) = *R*_0_ and *M*(0) = *M*_0_) of the shear load and bending moment functions. Substituting these conditions into Equations (15) and (16), we obtain *C*_1_ = *C*_2_ = 0.

The bending moment functions for each one of the four sections of the multilayered microresonator are determined from Equation (16):

For 0 < *x* < *L*_*S*_1__

(17a) $$M_{S_{1}}(x)=-M_{0}{\langle x-0\rangle }^{0}+R_{0}{\langle x-0\rangle }^{1}-\frac{1}{2}\omega_{S_{1}}{\langle x-0\rangle }^{2}$$

For *L*_*S*_1__ < *x* < *L*_*S*_12__

(17b) $$M_{S_{2}}(x)=-M_{0}{\langle x-0\rangle }^{0}+R_{0}{\langle x-0\rangle }^{1}-\frac{1}{2}\omega_{S_{1}}{\langle x-0\rangle }^{2}+\frac{1}{2}\omega_{S_{1}}{\langle x-L_{S_{1}}\rangle }^{2}-\frac{1}{2}\omega_{S_{2}}{\langle x-L_{S_{1}}\rangle }^{2}$$

For *L*_*S*_12__ < *x* < *L*_*S*_123__

(17c) $$\begin{array}{lll} M_{S_{3}}(x) & = & -M_{0}{\langle x-0\rangle }^{0}+R_{0}{\langle x-0\rangle }^{1}-\frac{1}{2}\omega_{S_{1}}{\langle x-0\rangle }^{2}+\frac{1}{2}\omega_{S_{1}}{\langle x-L_{S_{1}}\rangle }^{2}\\ & & -\frac{1}{2}\omega_{S_{2}}{\langle x-L_{S_{1}}\rangle }^{2}+\frac{1}{2}\omega_{S_{2}}{\langle x-L_{S_{12}}\rangle }^{2}-\frac{1}{2}\omega_{S_{3}}{\langle x-L_{S_{12}}\rangle }^{2} \end{array}$$

For *L*_*S*_123__ < *x* < *L*_*S*_1234__

(17d) $$\begin{array}{lll} M_{S_{4}}(x) & = & -M_{0}{\langle x-0\rangle }^{0}+R_{0}{\langle x-0\rangle }^{1}-\frac{1}{2}\omega_{S_{1}}{\langle x-0\rangle }^{2}+\frac{1}{2}\omega_{S_{1}}{\langle x-L_{S_{1}}\rangle }^{2}-\frac{1}{2}\omega_{S_{2}}{\langle x-L_{S_{1}}\rangle }^{2}\\ & & +\frac{1}{2}\omega_{S_{2}}{\langle x-L_{S_{12}}\rangle }^{2}-\frac{1}{2}\omega_{S_{3}}{\langle x-L_{S_{12}}\rangle }^{2}+\frac{1}{2}\omega_{S_{3}}{\langle x-L_{S_{123}}\rangle }^{2}-\frac{1}{2}\omega_{S_{4}}{\langle x-L_{S_{123}}\rangle }^{2} \end{array}$$

Then, the deflection *y_Sj_*(*x*) in each section of the multilayered microresonator is determined by substituting Equation (17) into Equation (9) and considering the boundary conditions expressed by Equation (10), as well as the integration rules of the Macaulay’s functions. Thus, the deflections *y_Sj_*(*x*) can be determined as:

For 0 < *x* < *L*_*S*_1__

(18a) $$y_{S_{1}}(x)=\frac{1}{{\left(EI_{z}\right)}_{S_{1}}}\left[-\frac{1}{2}M_{0}{\langle x-0\rangle }^{2}+\frac{1}{6}R_{0}{\langle x-0\rangle }^{3}-\frac{1}{24}\omega_{S_{1}}{\langle x-0\rangle }^{4}\right]$$

For *L*_*S*_1__ < *x* < *L*_*S*_12__

(18b) $$\begin{array}{lll} y_{S_{2}}(x) & = & \frac{1}{{\left(EI_{z}\right)}_{S_{2}}}[-\frac{1}{2}M_{0}{\langle x-0\rangle }^{2}+\frac{1}{6}R_{0}{\langle x-0\rangle }^{3}-\frac{1}{24}\omega_{S_{1}}{\langle x-0\rangle }^{4}+\frac{1}{24}\omega_{S_{1}}{\langle x-L_{S_{1}}\rangle }^{4}-\frac{1}{24}\omega_{S_{2}}{\langle x-L_{S_{1}}\rangle }^{4}\\ & & -\frac{1}{2}M_{0}L_{S_{1}}^{2}+\frac{1}{6}R_{0}L_{S_{1}}^{3}-\frac{1}{8}\omega_{S_{1}}L_{S_{1}}^{4}+\left(M_{0}L_{S_{1}}-\frac{1}{2}R_{0}L_{S_{1}}^{2}+\frac{1}{6}\omega_{S_{1}}L_{S_{1}}^{3}\right)x]+\frac{1}{{\left({\mathrm{EI}}_{z}\right)}_{S_{1}}}[(M_{0}L_{S_{1}}+\frac{1}{2}R_{0}L_{S_{1}}^{2}\\ & & -\frac{1}{6}\omega_{S_{1}}L_{S_{1}}^{3})x+\frac{1}{2}M_{0}L_{S_{1}}^{2}-\frac{1}{3}R_{0}L_{S_{1}}^{3}+\frac{1}{8}\omega_{S_{1}}L_{S_{1}}^{4}] \end{array}$$

For *L*_*S*_12__ < *x* < *L*_*S*_123__

(18c) $$\begin{array}{ll} y_{S_{3}}(x) & =\frac{1}{{\left(EI_{z}\right)}_{S_{3}}}[-\frac{1}{2}M_{0}{\langle x-0\rangle }^{2}+\frac{1}{6}R_{0}{\langle x-0\rangle }^{3}-\frac{1}{24}\omega_{S_{1}}{\langle x-0\rangle }^{4}+\frac{1}{24}\omega_{S_{1}}{\langle x-L_{S_{1}}\rangle }^{4}\\ & \;\;-\frac{1}{24}\omega_{S_{2}}{\langle x-L_{S_{1}}\rangle }^{4}+\frac{1}{24}\omega_{S_{4}}{\langle x-L_{S_{12}}\rangle }^{4}-\frac{1}{24}\omega_{S_{3}}{\langle x-L_{S_{12}}\rangle }^{4}+C_{3}x+C_{4}]\\ & \;\;C_{3}=\frac{{\left({\mathrm{EI}}_{z}\right)}_{S_{3}}}{{\left({\mathrm{EI}}_{z}\right)}_{S_{2}}}\left[-M_{0}L_{S_{2}}+\frac{1}{2}R_{0}L_{S_{2}}(2L_{S_{1}}+L_{S_{2}})-\frac{1}{2}\omega_{S_{1}}L_{S_{1}}L_{S_{2}}L_{S_{12}}-\frac{1}{6}\omega_{S_{2}}L_{S_{2}}^{3}\right]\\ & \;\;\;\;+\frac{{\left({\mathrm{EI}}_{z}\right)}_{S_{3}}}{{\left({\mathrm{EI}}_{z}\right)}_{S_{1}}}\left[-M_{0}L_{S_{1}}+\frac{1}{2}R_{0}L_{S_{1}}-\frac{1}{6}\omega_{S_{1}}L_{S_{1}}^{3}\right]\\ & \;\;\;\;+M_{0}L_{S_{12}}-\frac{1}{2}R_{0}L_{S_{12}}^{2}+\frac{1}{6}\omega_{S_{1}}L_{S_{1}}(L_{S_{1}}^{2}+3L_{S_{1}}L_{S_{2}}+3L_{S_{2}}^{2})+\frac{1}{6}\omega_{S_{2}}L_{S_{2}}^{3}\\ C_{4} & =\frac{{\left({\mathrm{EI}}_{z}\right)}_{S_{3}}}{{\left({\mathrm{EI}}_{z}\right)}_{S_{2}}}[\frac{1}{2}M_{0}L_{S_{2}}(2L_{S_{1}}+L_{S_{2}})-\frac{1}{3}R_{0}L_{S_{2}}\left[3L_{S_{1}}L_{S_{12}}+L_{S_{2}}^{2}\right]+\frac{1}{12}\omega_{S_{1}}L_{S_{1}}L_{S_{2}}\left[3L_{S_{1}}(2L_{S_{1}}+3L_{S_{2}})+4L_{S_{2}}^{2}\right]\\ & \;\;+\frac{1}{24}\omega_{S_{2}}L_{S_{2}}^{3}(4L_{S_{1}}+3L_{S_{2}})]+\frac{{\left({\mathrm{EI}}_{z}\right)}_{S_{3}}}{{\left({\mathrm{EI}}_{z}\right)}_{S_{1}}}\left[\frac{1}{2}M_{0}L_{S_{1}}^{2}-\frac{1}{3}R_{0}L_{S_{1}}^{3}+\frac{1}{8}\omega_{S_{1}}L_{S_{1}}^{4}\right]-\frac{1}{2}M_{0}L_{S_{12}}^{2}+\frac{1}{3}R_{0}L_{S_{12}}^{3}\\ & \;\;-\frac{1}{24}\omega_{S_{1}}L_{S_{1}}\left[3L_{S_{1}}(L_{S_{1}}^{2}+4L_{S_{1}}L_{S_{2}}+6L_{S_{2}}^{2})+8L_{S_{2}}^{3}\right]-\frac{1}{24}\omega_{S_{2}}L_{S_{2}}^{3}(4L_{S_{1}}+3L_{S_{2}}) \end{array}.$$

For *L*_*S*_123__ < *x* < *L*_*S*_1234__

(18d) $$\begin{array}{ll} y_{S_{4}}(x) & =\frac{1}{{\left({\mathrm{EI}}_{z}\right)}_{S_{4}}}{[-\frac{1}{2}M_{0}\langle x-0\rangle }^{2}+\frac{1}{6}R_{0}{\langle x-0\rangle }^{3}-\frac{1}{24}\omega_{S_{1}}{\langle x-0\rangle }^{4}+\frac{1}{24}\omega_{S_{1}}{\langle x-L_{S_{1}}\rangle }^{4}-\frac{1}{24}\omega_{S_{2}}{\langle x-L_{S_{1}}\rangle }^{4}\\ & \;\;\;+\frac{1}{24}\omega_{S_{2}}{\langle x-L_{S_{12}}\rangle }^{4}-\frac{1}{24}\omega_{S_{3}}{\langle x-L_{S_{12}}\rangle }^{4}+\frac{1}{24}\omega_{S_{3}}{\langle x-L_{S_{123}}\rangle }^{4}-\frac{1}{24}\omega_{S_{4}}{\langle x-L_{S_{123}}\rangle }^{4}+C_{5}x+C_{6}]\\ & C_{5}=\frac{{\left(EI_{z}\right)}_{S_{4}}}{{\left({\mathrm{EI}}_{z}\right)}_{S_{3}}}\left[-M_{0}L_{S_{3}}+\frac{1}{2}R_{0}L_{S_{3}}(2L_{S_{12}}+L_{S_{3}})-\frac{1}{2}\omega_{S_{1}}L_{S_{1}}L_{S_{3}}(L_{S_{1}}+2L_{S_{2}}+L_{S_{3}})-\frac{1}{2}\omega_{S_{2}}L_{S_{2}}L_{S_{3}}(L_{S_{2}}+L_{S_{3}})\right]\\ & \;\;\;\;+\frac{{\left({\mathrm{EI}}_{z}\right)}_{S_{4}}}{{\left({\mathrm{EI}}_{z}\right)}_{S_{2}}}\left[-M_{0}L_{S_{2}}+\frac{1}{2}R_{0}L_{S_{2}}(2L_{S_{1}}+L_{S_{2}})-\frac{1}{2}\omega_{S_{1}}L_{S_{1}}L_{S_{2}}L_{S_{12}}-\frac{1}{6}\omega_{S_{2}}L_{S_{2}}^{3}\right]\\ & \;\;\;\;+\frac{{\left({\mathrm{EI}}_{z}\right)}_{S_{4}}}{{\left({\mathrm{EI}}_{z}\right)}_{S_{1}}}\left[-M_{0}L_{S_{1}}+\frac{1}{2}R_{0}L_{S_{1}}^{2}-\frac{1}{6}\omega_{S_{1}}L_{S_{1}}^{3}\right]+M_{0}L_{S_{123}}-\frac{1}{2}R_{0}L_{S_{123}}^{2}\\ & \;\;\;\;+\frac{1}{6}\omega_{S_{1}}L_{S_{1}}\left[L_{S_{1}}(L_{S_{1}}+3L_{S_{2}}+3L_{S_{3}})+3L_{S_{2}}(L_{S_{2}}+2L_{S_{3}})+3L_{S_{3}}^{2}\right]\\ & \;\;\;\;+\frac{1}{6}\omega_{S_{2}}L_{S_{2}}\left[L_{S_{2}}(L_{S_{2}}+3L_{S_{3}})+3L_{S_{3}}^{2}\right]+\frac{1}{6}\omega_{S_{3}}L_{S_{3}}^{2}\\ & C_{6}=\frac{{\left({\mathrm{EI}}_{z}\right)}_{S_{4}}}{{\left({\mathrm{EI}}_{z}\right)}_{S_{3}}}[-M_{0}L_{S_{3}}(2L_{S_{12}}+L_{S_{3}})-\frac{1}{3}R_{0}L_{S_{3}}\left[3L_{S_{1}}(L_{S_{1}}+2L_{S_{2}}+L_{S_{3}})+3L_{S_{2}}(L_{S_{2}}+L_{S_{3}})+L_{S_{3}}^{2}\right]\\ & \;\;\;\;+\frac{1}{12}\omega_{S_{1}}L_{S_{1}}L_{S_{3}}\left[3L_{S_{1}}(2L_{S_{1}}+6L_{S_{2}}+3L_{S_{3}})+12L_{S_{2}}(L_{S_{2}}+L_{S_{3}})+4L_{S_{3}}^{2}\right]-\frac{1}{24}\omega_{S_{3}}L_{S_{3}}^{4}\\ & \;\;\;\;+\frac{1}{12}\omega_{S_{2}}L_{S_{2}}L_{S_{3}}\left[6L_{S_{1}}(L_{S_{2}}+L_{S_{3}})+3L_{S_{2}}(2L_{S_{2}}+3L_{S_{3}})+4L_{S3}^{2}\right]]+\frac{{\left(EI_{z}\right)}_{S_{4}}}{{\left({\mathrm{EI}}_{z}\right)}_{S_{2}}}[\frac{1}{2}M_{0}L_{S_{2}}(2L_{S_{1}}+L_{S_{2}})\\ & \;\;\;\;-\frac{1}{3}R_{0}L_{S_{2}}(3L_{S_{1}}L_{S_{12}}+L_{S_{2}}^{2})+\frac{1}{12}\omega_{S_{1}}L_{S_{1}}L_{S_{2}}\left[3L_{S_{1}}(2L_{S_{1}}+3L_{S_{2}})\right]+\frac{1}{24}\omega_{S_{2}}L_{S_{2}}^{3}(4L_{S_{1}}+3L_{S_{2}})]\\ & \;\;\;\;+\frac{{\left({\mathrm{EI}}_{z}\right)}_{S_{4}}}{{\left({\mathrm{EI}}_{z}\right)}_{S_{1}}}\left[\frac{1}{2}M_{0}L_{S_{1}}^{2}-\frac{1}{3}R_{0}L_{S_{1}}^{3}+\frac{1}{8}\omega_{S_{1}}L_{S_{1}}^{4}\right]-\frac{1}{2}M_{0}L_{S_{123}}^{2}+\frac{1}{3}R_{0}L_{S_{123}}^{3}-\frac{1}{24}\omega_{S_{3}}L_{S_{3}}^{3}(4L_{S_{12}}+3L_{S_{3}})\\ & \;\;\;\;-\frac{1}{24}\omega_{S_{1}}L_{S_{1}}\left[3L_{S_{1}}(L_{S_{1}}^{2}+4L_{S_{1}}L_{S_{2}}+4L_{S_{1}}L_{S_{3}}+12L_{S_{2}}L_{S_{3}}+6L_{S_{2}}^{2}+6L_{S_{3}}^{2})+8L_{S_{2}}(L_{S_{2}}^{2}+3L_{S_{2}}L_{S_{3}}+3L_{S_{3}}^{2})+8L_{S_{3}}^{3}\right]\\ & \;\;\;\;-\frac{1}{24}\omega_{S_{2}}L_{S_{2}}\left[4L_{S_{1}}(L_{S_{2}}^{2}+3L_{S_{2}}L_{S_{3}}+3L_{S_{3}}^{2})+3L_{S_{2}}(L_{S_{2}}^{2}+4L_{S_{2}}L_{S_{3}}+6L_{S_{3}}^{2})+8L_{S_{3}}^{3}\right] \end{array}.$$

For multilayered microresonators with two or three different sections, only the first two or three deflections equations must be used. Next, *y_Sj_*(*x*) is used into Equations (6) and (7) to find the maximum potential and kinetic energy of the microresonator. Finally, by substituting these energies into Equation (8), the first bending resonant frequency of the multilayered microresonator can be estimated.

## 3. Application of the Analytical Model

We applied our analytical model to estimate the first bending resonant frequency and deflection of two multilayered microresonators reported in the literature. In addition, we used finite element models (FEMs) to study the bending vibration of these microresonators. Our analytical results agree with the FEMs and experimental results of the two microresonators.

**Figure 5.**
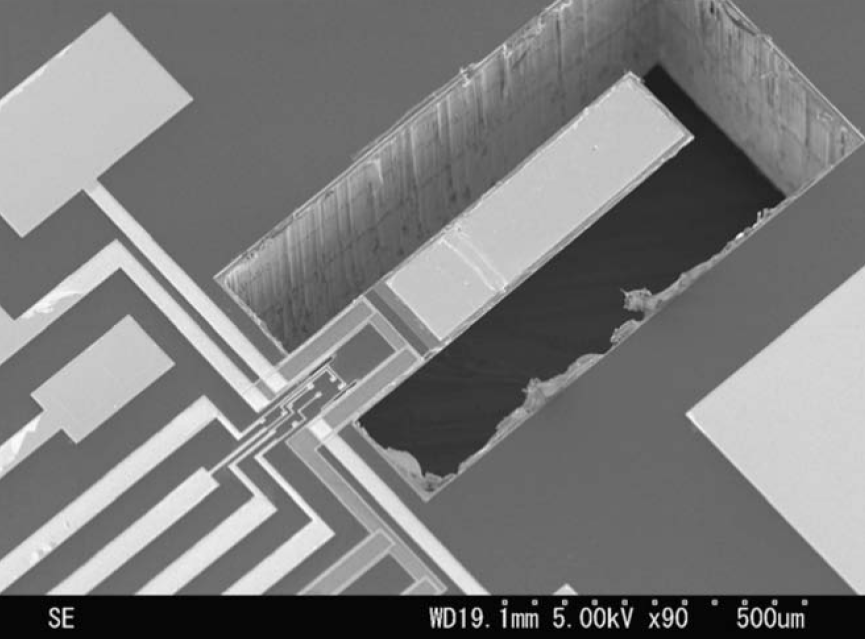
SEM micrograph of the multilayered microresonator type-A [46]. Reprinted with permission from Elsevier Science B.V. Copyright© 2009.

**Figure 6.**
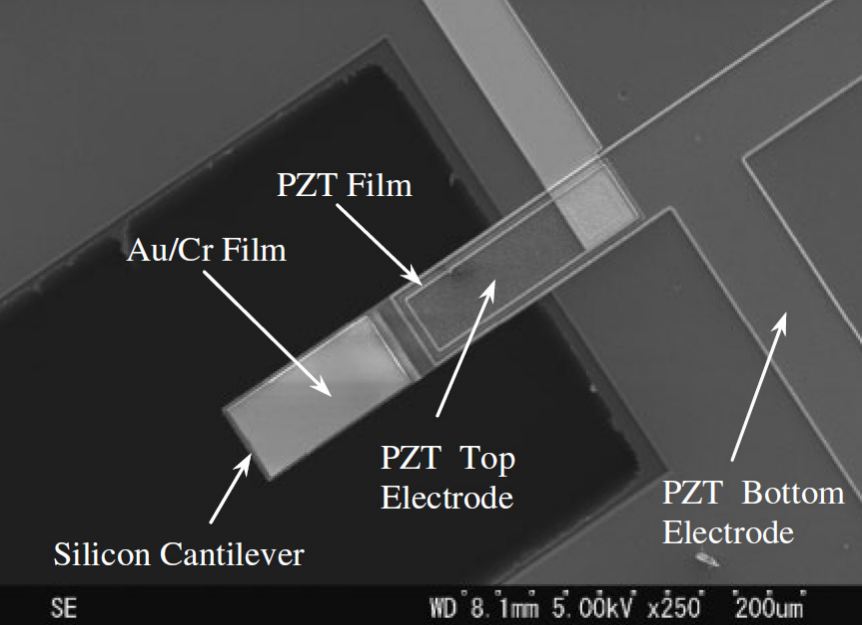
SEM micrograph of the multilayered microresonator type-B [47]. Reprinted with permission from Japan Society of Applied Physics. Copyright© 2007.

We considered the geometrical configuration of two multilayered microresonators for mass sensing applications developed by Lu *et al*. [46,47]. Figure 5 shows a SEM image of the first multilayered microresonator of Lu *et al*. [46], which we called microresonator type-A. In addition, we named their second multilayered microresonator microresonator type-B (see Figure 6).

**Figure 7.**
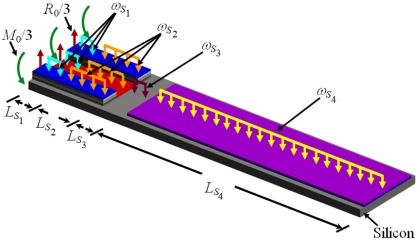
Geometrical configuration of the layers and load types on the microresonator type-A. This figure is not drawn in scale.

**Figure 8.**
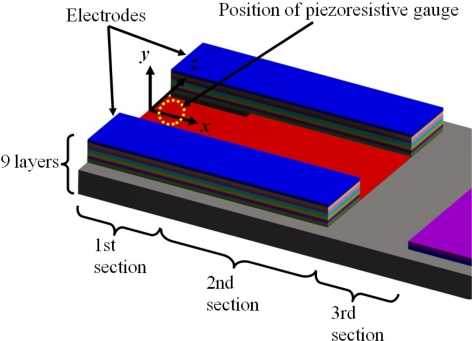
Detail view of the layers located on the first three sections of the microresonator type-A. This figure is not drawn in scale.

These microresonators have self-actuation and self-sensing capability. The details of their fabrication process can be found elsewhere [46,47]. Figure 7 shows the geometrical configuration of the layers and load types on the microresonator type-A. It uses small electrodes of piezoelectric lead zirconate titanate (PZT) for microresonator excitation and contains a piezoresistive gauge for detection of its resonant frequency (see Figure 8). It can be divided in four different sections of layers. The first two sections contain nine layers composed by the following materials: silicon (Si), silicon dioxide (SiO_2_), titanium (Ti), platinum (Pt), PZT, chromium (Cr), and gold (Au). The order of the nine layers on the first and second section is the following: Si/SiO_2_/Ti/Pt/PZT/Cr/Au/Cr/SiO_2_. Only the first section has two holes to decrease initial stress due to PZT deformation, which improve the signal to noise of the piezoresistive gauge contained in the first section. The third only contains a Si layer and the fourth section has three layers of Si/Cr/Au.

**Figure 9.**
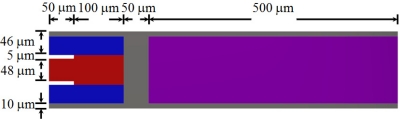
Detail view of the layers located on the first three sections of the microresonator type-A. This figure is not drawn in scale.

Table 1 indicates the order of the layers in each one of the four sections for the microresonator type-A. Figure 9 depicts the main dimensions of the microresonator type-A. In addition, Table 2 indicates the geometrical parameters of all layers used in the microresonator type-A. For the first section, *b*_*iS*1_ include the overall width of the layers located to a same distance *h*_*iS*1_.

**Figure 10.**
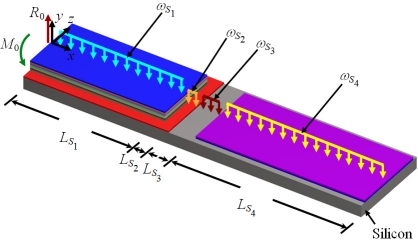
Geometrical configuration of the layers and load types on the microresonator type-B. This figure is not drawn in scale.

**Figure 11.**
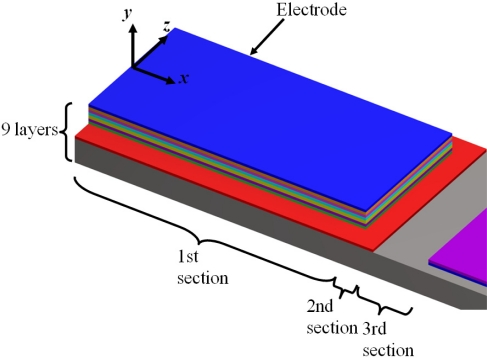
Detail view of the layers located on the first three sections of the microresonator type-B. This figure is not drawn in scale.

Figure 10 shows the geometrical configuration of the layers and load types on the microresonator type-B, which contains four different sections of layers. Its first section is integrated by nine layers that are fabricated with the same materials used in the first section of the microresonator type A. The second section is composed by two layers (Si/SiO_2_) and the third section is only formed by Si (see Figure 11). The fourth section contains three layers that are made of Si/Cr/Au. The order of the layers in each one of the four sections for the microresonator type-B is showed in Table 3. Table 4 indicates the geometrical parameters of all layers employed in the microresonator type-B.

Both microresonator type-A and type-B have four sections with different numbers of layers in each one of them. In addition, their layers have a symmetrical configuration with respect to the *xy*−plane. Therefore, our analytical model for a general multilayered microresonator can be used to estimate the first bending resonant frequency of both microresonator type-A and type-B. Table 5 shows the mechanical properties of all layers used in the two microresonators, which were obtained from the literature [48–51]. Based on the mechanical properties and values of the geometrical parameters of the two microresonators, we first find the weight by unit length, reaction load, bending moment, and effective stiffness for both microresonators (see Table 6). Using these values, we substitute the deflections Equation (18) into Equations (6) and (7) in order to determine the maximum potential and kinetic energies. Finally, these energies were applied into Equation (8) to estimate the first bending resonant frequency (*f_r_*) of the microresonators. Thus, our analytical results for the microresonator type-A and type-B are 13.18 kHz and 186.51 kHz, respectively. These results agree well with the experimental results of both microresonators obtained by Lu *et al*. [46,47], which are 12.81 kHz and 186.0 kHz, respectively. Our analytical results present a small relative difference of 2.9% and 0.3% with respect to experimental data. Also, we compared our analytical results with those obtained through two FEMs and a simple cantilever model (SCM).

For a simple cantilever model where the origin of its coordinate system is located at its fixed end and the *xz*–plane is considered its neutral plane, the first bending resonant frequency (*f_rc_*) can be calculated as [52]:

(19) $$f_{\mathrm{rc}}=0.162\frac{h_{c}}{L_{c}^{2}}\sqrt{\frac{E_{c}}{\rho_{c}}}$$

where *h_c_* and *L_c_* are the thickness and length of the cantilever, respectively; *E_c_* and *ρ_c_* are the Young’s modulus and density of the cantilever material.

The deflection curve (*y_c_*(*x*)) for a simple cantilever model can be determined as [53]:

(20) $$y_{c}(x)=\frac{\omega_{c}}{24E_{c}I_{c}}(x^{2}-4L_{c}x+6L_{c}^{2})$$

where *ω_c_* and *I_c_* are the weight by unit length and second moment of area of the cantilever.

Both microresonators type-A and type-B can be approximated to a simple cantilever model if only a layer in each one of their four sections is considered. For this, we used the layer with the largest thickness, which corresponds to the silicon layer for both microresonators. For the microresonator type-A, we neglected the holes and considered a silicon layer with dimensions 700 × 150 × 5 μm. This layer presents dimensions much larger than any other layer of the microresonator type-A. For the microresonator type-B, the dimensions (80 × 38 × 1 μm) of its piezoelectric layer (PZT) are close to those of its silicon layer (200 × 50 × 5.25 μm). Using Equation (19), the first bending resonant frequencies for both microresonators are 12.35 kHz and 158.82 kHz, respectively. For the microresonator type-A, the SCM’s result has a small relative difference of −3.6% with respect to experimental result. For this case, the approximation of the microresonator to a SCM is suitable because the microresonator’s dimensions are close to dimensions of its silicon layer (*i.e.*, the piezoelectric and isolation layers have dimensions much smaller than those of the silicon layer). Nevertheless, the SCM’s result for the microresonator type-B has a high relative difference of −14.6% with respect to the measured frequency. This high relative difference is caused because both metallic and isolation layers have an overall thickness and length (2 μm and 80 μm) close to those of the silicon layer (5.25 μm and 200 μm). Therefore, the mass and stiffness of the metallic and isolation layers have a higher influence on the mass and effective stiffness of the microresonator. For this case, our analytical model is more suitable than the SCM.

In addition, we made two FEMs for both microresonators through ANSYS^®^ software. These models used solid95 type elements, in which each element is defined by 20 nodes with three degrees of freedom per node: translations in the nodal *x*, *y*, and *z* directions. For FEM of the microresonator type-A, we only considered its thicker layers (silicon, SiO_2_, and PZT) in order to obtain a mesh that does not overcome the maximum nodes number (125,000 nodes) allowed by our ANSYS^®^ software license.

**Figure 12.**
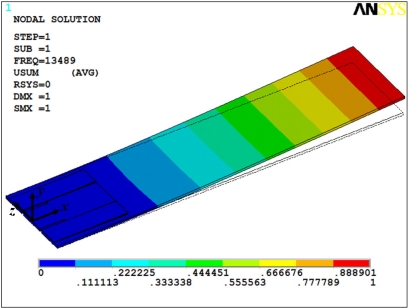
First bending mode of the microresonator type-A obtained using a FEM.

**Figure 13.**
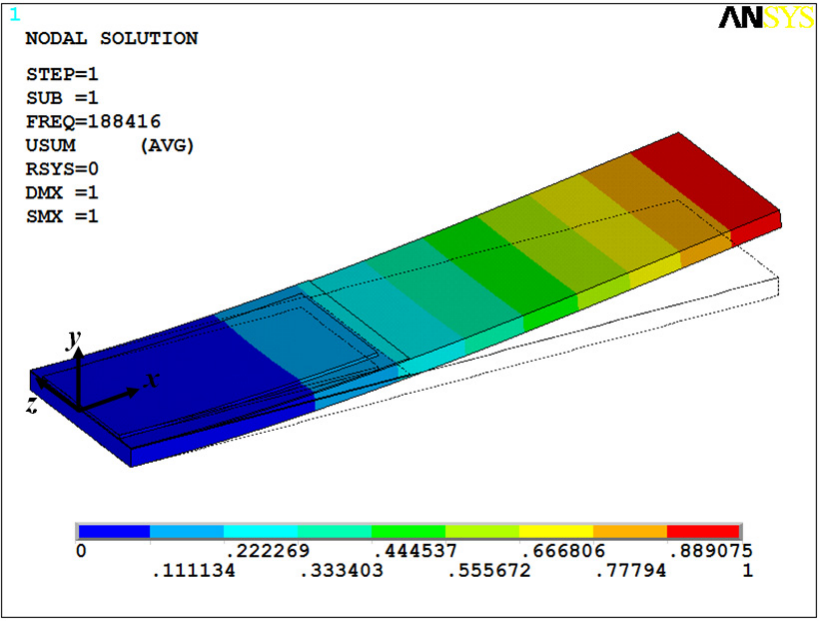
First bending mode of the microresonator type-B obtained using a FEM.

The thinner layers of the microresonator type-A have thicknesses less than 300 nm, which significantly increases the number of nodes in the FEM mesh. This is a problem to mesh layers on the order of nanometers. Similarly, the FEM of the microresonator type-B only included the thicker layers (silicon, SiO_2_, and PZT layers). Figures 12 and 13 show the first bending vibration mode of the FEMs, which were obtained to 13.49 kHz and 188.42 kHz. These results have a relative difference of 5.3% and 1.3% with respect to the experimental results.

**Figure 14.**
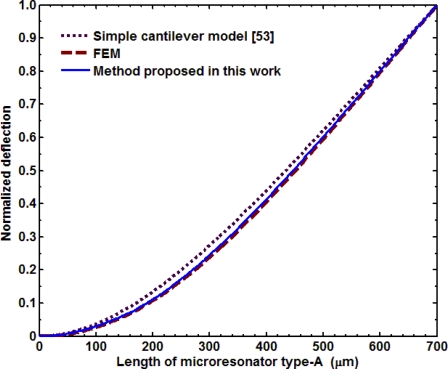
Normalized deflection *y*(*x*)/*y*_max_ of the microresonator type-A obtained through our analytical model, a FEM, and a simple cantilever model.

**Figure 15.**
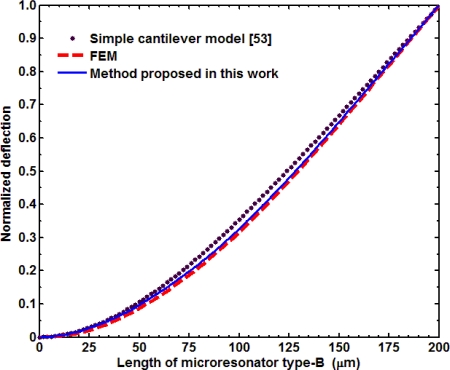
Normalized deflection *y*(*x*)/*y*_max_ of the microresonator type-B obtained through our analytical model, a FEM, and a simple cantilever model.

In addition, we compared the normalized deflection *y*(*x*)/*y_max_* of both microresonators obtained by the three models studied in this work. For this, we used the deflections Equations (18) and (20), dividing their results by the maximum deflection. Figures 14 and 15 illustrate the normalized deflections along the length of both microresonators, which were obtained through the SCM, FEMs, and our analytical model. The results of normalized deflections obtained by our analytical model agree very well with those of the FEMs.

Our analytical model can be used in the design phase of multilayered microresonators with variable cross section in order to estimate their lowest bending resonant frequencies and deflections. Using our model, a designer can determine the dimensions of the microresonator’s layers that allow it to operate at a resonant frequency suitable to a particular application. A designer can also use the proposed model to know the influence of the microresonator’s materials on its resonant frequency.

## 4. Conclusions

An analytical model to estimate the first bending resonant frequency and deflection curve of multilayered microresonator with variable cross-section was presented. This model is formulated through the Rayleigh and Macaulay methods, as well as the Euler-Bernoulli beam theory. We have applied our analytical model to two multilayered microresonators composed by layers of seven different materials reported in the literature. The results of proposed analytical model presented a relative difference less than 3.0% with respect to experimental data. Our analytical model can be useful in the mechanical design of multilayered microresonator for detection of mass and chemical species. Future work will include the effect of the residual stress on the multilayered microresonators with variable cross-section. In addition, we will study the resonant frequency shift of these microresonators due to films or particles deposited on their surface.

## Figures and Tables

**Table 1.** Order of the layers in the four sections of the microresonator type-A.

**Layer**	**Section 1**	**Section 2**	**Section 3**	**Section 4**

Si	1st	1st	1st	1st
SiO_2_	2nd	2nd		
Ti	3rd	3rd		
Pt	4th	4th		
PZT	5th	5th		
Cr	6th	6th		2nd
Au	7th	7th		3rd
Cr	8th	8th		
SiO_2_	9th	9th		

**Table 2.** Geometrical parameters of the layers used in the microresonator type-A.

**Geometrical parameter**	**Dimension (μm)**	**Geometrical parameter**	**Dimension (μm)**

*L*_*S*1_ = *L*_*S*3_	50	*b*_2*S*3_ = *b*_3*S*4_	130
*L*_*S*2_	100	*t*_1*S*1_ = *t*_1*S*2_ = *t*_1*S*3_ = *t*_1*S*4_	5.0
*L*_*S*4_	500	*t*_2*S*1_ = *t*_2*S*2_	0.40
*b*_1S1_	140	*t*_3*S*1_ = *t*_3*S*2_	0.05
*b*_2S1_	120	*t*_4*S*1_ = *t*_4*S*2_	0.25
*b*_3*S*1_ = *b*_4S1_ = *b*_5S1_ = *b*_6S1_	72	*t*_5*S*1_ = *t*_5*S*2_	1.0
*b*_7*S*1_ = *b*_8*S*1_ = *b*_9*S*1_ = *b*_3*S*2_	72	*t*_6*S*1_ = *t*_6*S*2_ = *t*_8*S*1_ = *t*_8*S*2_	0.03
*b*_4*S*2_ = *b*_5*S*2_ = *b*_6*S*2_ = *b*_7*S*2_	72	*t*_7*S*1_ = *t*_7*S*2_ = *t*_3*S*4_	0.15
*b*_8*S*2_ = *b*_9*S*2_	72	*t*_9*S*1_ = *t*_9*S*2_	0.30
*b*_1*S*3_ = *b*_1*S*4_	150		

**Table 3.** Order of the layers in the four sections of the microresonator type-B.

**Layer**	**Section 1**	**Section 2**	**Section 3**	**Section 4**

Si	1st	1st	1st	1st
SiO_2_	2nd	2nd		
Ti	3rd			
Pt	4th			
PZT	5th			
Cr	6th			2nd
Au	7th			3rd
Cr	8th			
SiO_2_	9th			

**Table 4.** Geometrical parameters of the layers used in the microresonator type-B.

**Geometrical parameter**	**Dimension (μm)**	**Geometrical parameter**	**Dimension (μm)**

*L*_*S*1_	80	*h*_1*S*1_ = *h*_1*S*2_ = *h*_1*S*3_ = *h*_1*S*4_	5.25
*L*_*S*2_	6	*h*_2*S*1_ = *h*_2*S*2_	0.40
*L*_*S*3_	14	*h*_3*S*1_	0.05
*L*_*S*4_	100	*h*_4*S*1_	0.25
*b*_1*S*1_ = *b*_2*S*1_ = *b*_1*S*2_ = *b*_2*S*2_	50	*h*_5*S*1_	1.0
*b*_3*S*1_ = *b*_4*S*1_ = *b*_5*S*1_ = *b*_6*S*1_	38	*h*_6*S*1_ = *h*_8*S*1_ = *h*_2*S*4_	0.03
*b*_7*S*1_ = *b*_8*S*1_ = *b*_9*S*1_	38	*h*_7*S*1_	0.15
*b*_2*S*4_ = *b*_3*S*4_	44	*h*_9*S*1_	0.30
*b*_1*S*3_ = *b*_1*S*4_	50	*h*_3*S*4_	0.24

**Table 5.** Mechanical properties of the layers used in the microresonator type-A and microresonator type-B.

**Layer**	**Young’s modulus (GPa)**	**Density (kg**·**m^−3^)**	**Poisson ratio**

Si	130	2330	0.28
SiO_2_	73	2200	0.17
Ti	110	4510	0.32
Pt	168	21,400	0.39
PZT	63	7500	0.36
Cr	140	7190	0.21
Au	75	19,300	0.42

**Table 6.** Values of the weight by unit length, reaction load, bending moment, and effective stiffness for the microresonator type-A and microresonator type-B.

**Parameter**	**Microresonator type-A**	**Microresonator type-B**

*R*_0_ × 10^9^ N	15.90	1.96
*M*_0_ × 10^12^ N·m	5.24	0.17
*ω*_*S*1_ × 10^6^ N·m^−1^	29.09	12.79
*ω*_*S*2_ × 10^6^ N·m^−1^	30.32	6.43
*ω*_*S*3_ × 10^6^ N·m^−1^	17.14	6.00
*ω*_*S*4_ × 10^6^ N·m^−1^	21.11	8.09
(*EI_z_*)_*S*1_ × 10^12^ N·m^2^	344.98	159.86
(*EI_z_*)_*S*2_ × 10^12^ N·m^2^	361.38	89.57
(*EI_z_*)_*S*3_ × 10^12^ N·m^2^	203.12	78.38
(*EI_z_*)_*S*4_ × 10^12^ N·m^2^	216.24	85.56
